# Curses or Cures: A Review of the Numerous Benefits Versus the Biosecurity Concerns of Conotoxin Research

**DOI:** 10.3390/biomedicines8080235

**Published:** 2020-07-22

**Authors:** Walden E. Bjørn-Yoshimoto, Iris Bea L. Ramiro, Mark Yandell, J. Michael McIntosh, Baldomero M. Olivera, Lars Ellgaard, Helena Safavi-Hemami

**Affiliations:** 1 Department of Biomedical Sciences, University of Copenhagen, 2200 Copenhagen N, Denmark; walden@sund.ku.dk (W.E.B.-Y.); iris.ramiro@sund.ku.dk (I.B.L.R.); 2 Eccles Institute of Human Genetics, University of Utah, Salt Lake City, UT 84112, USA; myandell@genetics.utah.edu; 3 Utah Center for Genetic Discovery, University of Utah, Salt Lake City, UT 84112, USA; 4 School of Biological Sciences, University of Utah, Salt Lake City, UT 84112, USA; mcintosh.mike@gmail.com (J.M.M.); olivera@biology.utah.edu (B.M.O.); 5 George E. Whalen Veterans Affairs Medical Center, Salt Lake City, UT 84148, USA; 6 Department of Psychiatry, University of Utah, Salt Lake City, UT 84108, USA; 7 Department of Biology, Linderstrøm-Lang Centre for Protein Science, University of Copenhagen, 2200 Copenhagen N, Denmark; lellgaard@bio.ku.dk; 8 Department of Biochemistry, University of Utah, Salt Lake City, UT 84112, USA

**Keywords:** conotoxin, conopeptide, cone snail, venom, envenomations, fatalities, drugs, biosecurity, biomedicine

## Abstract

Conotoxins form a diverse group of peptide toxins found in the venom of predatory marine cone snails. Decades of conotoxin research have provided numerous measurable scientific and societal benefits. These include their use as a drug, diagnostic agent, drug leads, and research tools in neuroscience, pharmacology, biochemistry, structural biology, and molecular evolution. Human envenomations by cone snails are rare but can be fatal. Death by envenomation is likely caused by a small set of toxins that induce muscle paralysis of the diaphragm, resulting in respiratory arrest. The potency of these toxins led to concerns regarding the potential development and use of conotoxins as biological weapons. To address this, various regulatory measures have been introduced that limit the use and access of conotoxins within the research community. Some of these regulations apply to all of the ≈200,000 conotoxins predicted to exist in nature of which less than 0.05% are estimated to have any significant toxicity in humans. In this review we provide an overview of the many benefits of conotoxin research, and contrast these to the perceived biosecurity concerns of conotoxins and research thereof.

## 1. Introduction

This article is divided into four sections. In the introductory section we provide an overview of the definition and classification of conotoxins, their chemical and pharmacological diversity, and a brief history of the methodologies used for conotoxin discovery. In the second section we highlight measurable scientific and societal benefits of conotoxin research with a view toward the future. The third section addresses biosecurity concerns and past and current regulations of conotoxins. In this section we discuss fatalities resulting from cone snail envenomations, toxicity data of selected conotoxins, the potential misuse of conotoxins as biological weapons, and their fictional use as murder weapons in the literature and popular media. In the concluding remarks, we assess the effectiveness and justification of regulations and suggest revisions of some current regulatory measures.

### 1.1. Conotoxin Definition, Classification, and Discovery

**Figure 1:**
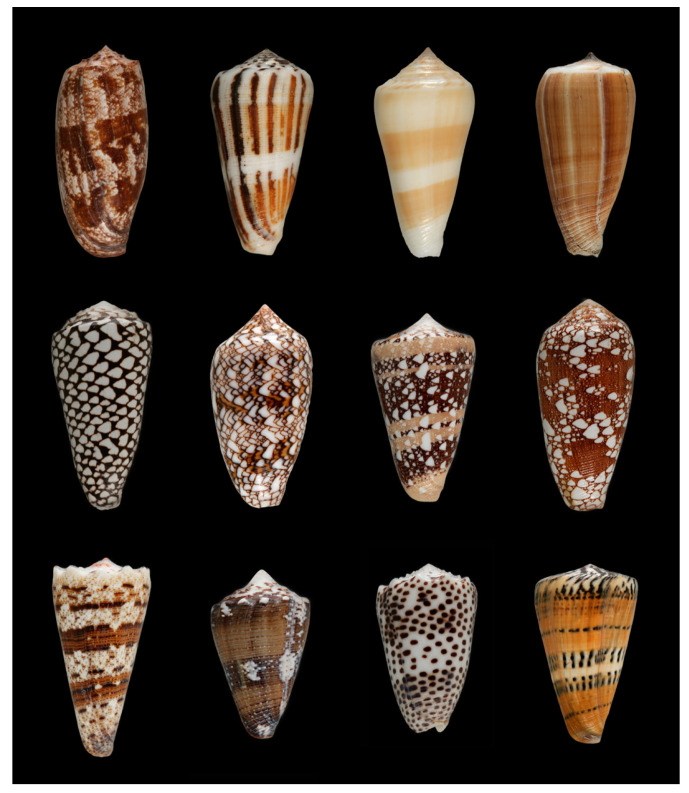
Shells of selected cone snail species from nine subgenera (for subgenus classification see [5]). Top row: fish-hunting cone snails (from left to right: *Conus geographus* (*Gastridium*), *Conus magus* and *Conus consors* (*Pionoconus*), *Conus* *purpurascens* (*Chelyconus*)), middle row: snail-hunting cone snails (*Conus marmoreus* (*Conus*), *Conus textile* and *Conus ammiralis (Cylinder*), *Conus omaria* (*Darioconus*)), bottom row: worm-hunting species (*Conus imperialis* and *Conus regius* (*Stephanoconus*), *Conus pulicarius* (*Puncticulis*), *Conus mustelinus* (*Rhizoconus*)). Shells not to scale.

Venomous cone snails comprise a large and diverse lineage of marine gastropods within the family of Conidae (superfamily Conoidea) [1,2,3,4]. Based on molecular phylogenetic data, cone snails can be grouped into ≈57 distinct clades (or subgenera) [5], all of which use venom for prey capture (examples shown in Figure 1).

In the most basic sense, a conotoxin is a toxin identified from any of the ≈1000 living cone snails. The majority of conotoxins are gene-derived peptides that are synthesized at the ribosome and further processed in the endoplasmic reticulum (ER) and Golgi apparatus of the secretory cells of the venom gland. Small molecules of non-peptidic nature have also been isolated from cone snail venom. These have traditionally not been defined as “conotoxins”, but instead named according to their characteristic chemical structures (for example [6,7]). Cone snail small molecules have not been subject to regulation and will therefore not be further discussed in this review.

The majority of conotoxins identified to date contain disulfide bonds that are formed between cysteine residues to confer structural stability and resistance against proteolytic degradation [8]. However, not all conotoxins contain cysteines and it has been suggested that conotoxins should be classified into those that are cysteine-rich (i.e., containing more than one disulfide bond) and those that are cysteine-poor (i.e., containing only one or no disulfide bonds). The term “conopeptide” was suggested to describe the latter group. However, this distinction has not received traction in the field and both terms conotoxin and conopeptide are now being used interchangeably [9].

Three biochemical and pharmacological features have been used to broadly classify conotoxins into distinct groups: their pharmacological target and activity (typically designated by a Greek letter), their cysteine framework (designated by Roman numerals) and their gene superfamily (designated by Latin letters). For example, conotoxins **αA**-G**I** and **αM**-M**III**J both target the nicotinic acetylcholine receptor (nAChR) as represented by the Greek letter **α** but their genes and cysteine frameworks do not share any homology; one belongs to the **A** gene superfamily and has a type **I** cysteine framework while the other belongs to the **M** gene superfamily and has a type **III** cysteine framework. To date, more than 10 distinct pharmacological classes, 50 gene superfamilies, and 28 cysteine frameworks have been described [10], and more are likely to be discovered in the future.

The five best studied pharmacological classes of conotoxins all target ion channels expressed in the nervous and locomotor systems: **α** (inhibitors of nAChR), **ω** (inhibitors of voltage-gated calcium channels, VGCC), **κ** (inhibitors of voltage-gated potassium channels, VGKC), **μ** (inhibitors of voltage-gated sodium channels, VGSC), and **δ** (delayers of activation of voltage-gated sodium channels, VGSC) (Table 1). Not all pharmacological classes of conotoxins have a Greek letter designation. Instead, some have been named according to their sequence homology or similarity to other peptides (e.g., conopressins share sequence homology to vasopressin-oxytocin and coninsulins to insulin) or according to their phenotypic effect in mice (e.g., Conantokins, toxins that induce a sleep-like state in mice, were named after the Filipino word for sleep, “antok”) (Table 1).

### 1.2. Conotoxin Discovery

In the early days of conotoxin discovery, dating back to the 1960s, conotoxins were directly isolated from dissected venom, usually by bioassay-directed fractionation and sequencing (for example [11,25,26,27]). Thus, discovery was focused on the biological activity of a newly identified toxin, and as such, the toxin’s pharmacological activity and classification was usually determined. A common assay used to identify new toxins was by intracranial (IC; into the brain) or intraperitoneal (IP; into the abdominal cavity) injection of fractionated venom compounds into mice followed by observational recording [11,22,25,26,27,28,29,30]. Sequencing of active components required several rounds of purification from the complex venom mixture. As the conotoxins that elicited the most severe phenotypes when injected in mice could be more easily traced during purification, most conotoxins identified early on were those that were potently active in vertebrates and elicited severe effects such as seizures, shaking, paralysis, respiratory distress, or death [11,22,25,26,27,28,29,30]. Conotoxins that did not elicit a strong physiological response were not pursued or not reported (for example, see [31]). This may have resulted in the perception that most conotoxins have severe toxicity in vertebrates. Additionally, early studies predominantly focused on the venom of fish-hunting (piscovorous) cone snails. However, fish hunters constitute fewer than 20% of the total species diversity of cone snails [32]. The vast majority of cone snails prey on worms (vermivorous), and a small fraction of species prey on other mollusks (molluscivorous). Conotoxins isolated from piscovorous species are more likely to show toxicity in vertebrates than those isolated from vermivorous and molluscivorous species. Indeed, as conotoxin research expanded to the venoms of worm- and snail-hunting species and to more diverse sets of toxins from fish-hunters (e.g., α-conotoxins that target neuronal nAChRs, coninsulins, conopressins), the number of conotoxins with no or very low phenotypic activity in vertebrates steadily increased [10,33]. The vast majority of conotoxins isolated from venom to date have little to no toxicity in vertebrates.

The advent of genome sequencing in the 2000s dramatically changed how conotoxins could be identified; toxin sequences could now be readily deduced from genomic DNA or mRNA without the need to physically isolate toxins from venom. This led to a dramatic increase in the rate of conotoxin discovery; today more than 20,000 conotoxin sequences have been identified with thousands more anticipated to be sequenced in the coming years. The vast majority of these conotoxins have never been directly isolated from venom and their pharmacological activity remains unknown. For toxin sequences that share significant homology with toxins of known pharmacologies, activity can sometimes be predicted, but potencies and subtype selectivity profiles are difficult to predict. Activities of conotoxin sequences that do not share significant homology with known toxins are impossible to predict and, one may argue, these should not even be called conotoxins until a biological activity or presence in venom has been verified. To address this issue we previously proposed the usage of “conotoxin candidate” or “putative conotoxin” until future evidence can verify that a newly identified sequence indeed encodes a biologically active toxin (and is not merely predicted to do so) [34]. However, currently, there is no consensus in the field about how to best define newly identified conotoxin sequences.

While the difficulty of defining and classifying toxin sequences from large datasets has not been perceived as a limitation in the field of conotoxin research, the lack of a clear definition combined with the complexity of biological activities and toxicities has complicated the generation of well-reasoned regulations for research on, and access to, conotoxins (see Section 3.4).

## 2. Conotoxin “Cures”—Scientific and Societal Benefits of Conotoxin Research

### 2.1. The Conotoxin Drug Ziconotide (Tradename Prialt^®^)

ω-Conotoxin MVIIA (or ziconotide) is arguably the most famous conotoxin discovered to date. First isolated from the venom of the magician cone, *Conus magus*, at the University of Utah in 1982 [26], it was developed as a drug for the treatment of intractable pain by the biotech company Neurex Corp, approved by the United States Food and Drug Administration (FDA) in 2004, and marketed as Prialt^®^ (the primary alternative to morphine) (Table 2). The history of the discovery of ω-conotoxin MVIIA has recently been reviewed in more detail elsewhere [35]. Here, we focus on the initial scientific goals that led to the discovery of ω-MVIIA and the societal benefits of this conotoxin today.

ω-MVIIA was discovered as part of an initiative into understanding why the venom of fish-hunting cone snails could be paralytic. In fish, ω-MVIIA was found to block neuromuscular transmission at the presynaptic terminus by inhibiting a specific voltage-gated calcium channel [36,37]. However, in the early 1980s, calcium channels had not been defined at a molecular level and it was uncertain how many different voltage-gated calcium channels were present in the vertebrate nervous system. The isolation of ω-MVIIA and a related peptide from *Conus geographus*, ω-GVIA, provided key pharmacological tools to define different types of voltage-gated calcium channels. Both peptides were selective for a calcium channel subtype that had not previously been recognized (initially known as the N-type calcium channel, and later as Ca_v_2.2). While exploring the potential biomedical applications of ω-MVIIA, experiments conducted by Neurex Corp with a radiolabeled analog revealed specific binding to layers of the spinal cord dorsal horn previously established to be important for the perception of pain [38]. This finding paved the way for the subsequent development of ω-MVIIA as an analgesic [39].

The commercial drug Prialt^®^ is an exact synthetic copy of ω-MVIIA. When approved by the FDA in 2004, Prialt was a welcome addition to the repertoire of anesthesiologists as an agent with a non-opioid mechanism. Unlike opioids, Prialt does not cause addiction or respiratory depression, but at high doses can lead to other severe, albeit not fatal, side effects, including psychomotor effects ranging from mild ataxia and auditory hallucinations (typically completely reversible with a small dose reduction) to more debilitating ataxia and psychosis. Furthermore, because Prialt acts by targeting Ca_v_2.2 channels expressed in the central nervous system, it must be administered intrathecally using an implanted pump. This is an invasive and relatively costly procedure that has been a barrier to more widespread use. Thus, clinically, Prialt was often used a last resort. However, due to the lack of availability of effective, non-opioid therapeutics, recent guidelines now encourage the use of Prialt as a first-line agent in various pain conditions including neuropathic and nociceptive pain [40]. Furthermore, Prialt has been increasingly used in combination with an intrathecal opioid, exploiting the potentially synergistic effect of Prialt and opioids in the treatment of refractory chronic and cancer pain [41].

### 2.2. Conotoxin Drug Leads

In addition to the clinical development of ω-MVIIA several other conotoxins have been at various stages of development as drug leads for pain, epilepsy, heart disease, and diabetes (for recent reviews on these toxins see [35,42,43,44]). Table 2 provides an overview of these drug leads. Despite their promising therapeutic applications, none of these conotoxins has (yet) reached clinical approval. It is difficult to assess the underlying reasons for this because information on commercial developments of drug leads is typically not made accessible to the public when the development of a compound is discontinued (e.g., information on lack of efficacy in clinical trials, safety concerns, change in a company’s development program, demise of a company, intellectual property disputes, etc.). Where known, we list the current development status of conotoxin drug leads and the reason for why past development efforts were halted (Table 2).

Regardless of their drug development status, many of these toxins have become valuable pharmacological and biomedical tools for the study of signaling pathways important in health and disease.

### 2.3. Diagnostic Tool

One hallmark feature of conotoxins is their target specificity for closely related subtypes of receptors and ion channels. The selectivity profile of ω-conotoxin GVIA from the venom of *Conus geographus*, a homolog of the approved drug Prialt, led to its development as a diagnostic tool for Lambert–Eaton myasthenic syndrome (LEMS). LEMS is an autoimmune disorder, which results in muscle weakness, and is associated with lung cancer. LEMS is caused by the production of antibodies against presynaptic voltage-gated calcium channels (VGCCs), which results in the inhibition of acetylcholine release at the neuromuscular junction [45,46,47]. While it has historically been difficult to differentiate LEMS from symptomatically related disorders, in 1989 Sher and coworkers showed that antibodies against VGCCs produced in LEMS could immunoprecipitate ^125^I-ω-conotoxin GVIA-bound N-type VGCCs (Ca_v_2.2), which are elevated in about half of LEMS patients [45,48]. This laid the basis for a diagnostic radio immunoprecipitation assay to differentiate LEMS from similar disorders, such as myasthenia gravis. By labeling solubilized cell membrane expressing Ca_v_2.2 with ^125^I-labeled ω-conotoxin GVIA, and exposing this to LEMS patient serum, antibodies against Ca_v_2.2 can bind the receptor-conotoxin complex. These are then precipitated, and the radioactivity can then be detected, indicating that the patient serum contains Ca_v_2.2 antibodies. This diagnosis was later improved by the use of a different conotoxin that binds P/Q-type VGCCs (Ca_v_2.1), ω-conotoxin MVIIC. Antibodies against Ca_v_2.1 are elevated in about 85 % of LEMS patients [49,50]. Differentiating these disorders is critical for guiding clinical care [51]. The emergence of medically relevant diagnostic tools provides an important example for the societal benefits of conotoxin research.

### 2.4. Cosmetics

Similarly to botulinium toxin (Botox^®^), conotoxins that have myorelaxant properties can be developed as anti-wrinkle creams or injectable formulations. One such conotoxin is μ-CIIIC, originally isolated from the fish-hunting cone snail *Conus consors* as part of the European Commission-funded CONCO project (“CONCO: the cone snail genome project for health”). μ-CIIIC preferentially blocks the skeletal muscle sodium channel, Na_v_1.4, and the neuronal sodium channel Na_v_1.2 [52]. Due to the blocking of Na_v_1.4, it can act as a myorelaxant. μ-CIIIC was initially investigated as a drug for the treatment of pain and as a local anesthetic but is now sold as the active ingredient in a non-prescription cosmetic anti-wrinkle product under the name “XEP™-018”.

### 2.5. Research Tools

Conotoxins that target mammalian receptors are often selective for certain receptor subtypes, or subunit compositions. This feature renders conotoxins excellent tools for a plethora of studies in the areas of pharmacology, neuroscience, biochemistry, and structural biology. Table 3 lists a small number of these conotoxins, and examples of their use in basic biology and biomedical research. There is of course overlap with clinically developed conotoxins (Table 2), which are also often used as research tools. For instance, ω-conotoxin MVIIA (the drug Prialt), has been used as a tool compound in thousands of studies.

Another conotoxin that has been extensively used as a research tool in the scientific literature (> 3000 publications) is ω-conotoxin GVIA, a potent and selective blocker of the presynaptic N-type calcium channels, Ca_v_2.2. The Ca_v_2.2 channels play a crucial role in neurotransmitter release in response to action potentials in the kidneys, where they regulate the dilation of arteries, and in the heart, where they regulate cardiac excitability [53,54,55]. Hence, ω-conotoxin GVIA has been used extensively in numerous studies of various topics, including neurotransmission, pain, cardiology, epilepsy, renal function, and nuclear signaling (selected references in Table 3).

Another example is the α-conotoxin, ImI, from the vermivorous *Conus imperialis* (as well as the subsequently discovered α-conotoxin, ImII [56]). ImI and ImII are inhibitors of the neuronal α7 subtype of the nAChRs [57]. These toxins, like most other subtype- or subunit-selective conotoxins, have been used to elucidate the importance of receptor subunits in numerous biological- and pathophysiological studies [58,59,60]. However, they have also seen other more specialized uses. For instance, in a 2014 study Heghinian and co-workers used several different α-conotoxins to perform structurally guided mutations in the *D. melanogaster* α7 nAChR, allowing this receptor to display similar selectivity for various conotoxins as the mammalian counterpart. This, in turn, resulted in *D. melanogaster* cholinergic synapses that mimic the synaptic behavior of vertebrate synapses, improving the suitability of these mutant flies as a tool for in vivo drug discovery [61].

In a 2015 study, Lin and co-workers utilized the specificity of α-ImI for cellular targeting of the chemotherapy drug, paclitaxel [62]. The authors showed that linking paclitaxel-containing micelles to α-ImI significantly decreased the mass of tumors in mice when compared to either unlinked paclitaxel-filled micelles or free paclitaxel. In addition, they observed a lower systemic toxicity of the α-ImI-linked micelles.

In addition, several conotoxins have served as tools in structural biology to elucidate specific receptor binding sites or mechanisms of receptor activation. For instance, the X-ray crystal structure of the conotoxin con-ikot-ikot from *Conus striatus* [63] in complex with the GluR2 AMPA receptor subunit revealed the molecular mechanism underlying receptor activation [64]. Another example is the conotoxin Con-Insulin G1 from *Conus geographus* that revealed a minimum binding motif of insulin at the human insulin receptor [65].

Conotoxins undergo post-translational processing (folding and modification) in the ER and Golgi prior to packaging and secretion into the lumen of the venom gland. Due to their small size, chemical diversity, and high degree of post-translational modifications, conotoxins are ideal candidates to study general principles of peptide folding, modification, and secretion. Several conotoxins have been repeatedly used as model substrates for studies into enzyme-assisted peptide biosynthesis and folding, such as α-GI [66,67], μ-SmIIIA [68,69], and conantokin-G [70,71].

Lastly, conotoxins are among the most rapidly evolving gene products known in nature and have served as tools in a diverse range of studies on the effects of feeding ecology, prey taxa, dietary breadth, age and geographical heterogeneity on the evolution of venom genes [72,73,74,75,76], and studies on the role of gene duplication and positive selection on venom gene expression and diversification [77,78,79].

### 2.6. Conotoxin Research—A View toward the Future

Recent advances in throughput and sensitivity of next-generation DNA and peptide sequencing have resulted in a massive increase in the rate of conotoxin discovery (for example [34,113]). This is unlikely to decrease any time soon given that the cost of sequencing continues to fall. In combination with the generation of easier, less computationally heavy bioinformatic tools for data analysis, conotoxin discovery can now be done without the need of expensive or highly specialized equipment. The increasing rate of conotoxin discovery is being met with advances in methodologies for conotoxin production (for example [114,115,116]), high-content target screening and identification (for example [117,118,119]), and with a newly sparked interest in peptide-based drug development by the pharma industry [120]. We anticipate that this combination will lead to the development and design of many more conotoxin-based biomedical tools and pharmacological agents in the future.

## 3. Conotoxin “Curses”—Biosecurity Concerns

### 3.1. Cone Snail Envenomations and Human Fatalities

From the very first report of a human fatality from a cone snail sting ≈350 years ago, through to 2017, 141 cases of human envenomations have been recorded, of which 36 were fatal [121]. No human fatalities have been reported for the past 20 years. Most, if not all, of the 36 human fatalities caused by cone snail stings have been attributed to a single species, *Conus geographus* [121]. All of these were accidental, and there have been no reports of the use of cone snail venom as a weapon for murder.

In humans, symptoms from cone snail envenomations vary depending on several factors, including cone snail species. Often, pain or numbness is reported, but symptoms can include edema, vision impairment, fatigue and faintness, dyspnea, loss of reflexes, and nausea. Some victims have noticed a burning sensation at the site of the sting, while others have reported that the sting itself initially went unnoticed. Subsequently, reports of faintness, palpebral ptosis, dysphagia, as well as vision and speech impairment are common in more severe cases, though in some cases no obvious symptoms have been reported prior to the onset of muscle paralysis, which in the worst case can lead to death due to respiratory or cardiac arrest within a few hours [122,123,124]. No effective antivenom exists against cone snail venom.

While the venom of a small subset of the ≈800 species of cone snails is toxic to humans, the number of human envenomations by these animals pales in comparison to those reported for other venomous animals. Snake bites undoubtedly comprise the largest contribution of serious human envenomations by any group of animals. While exact data can be difficult to obtain, the World Health Organization estimates that ≈2.7 million people are envenomated by snakes every year, resulting in 81,000–138,000 deaths per year, and 400,000 permanent disabilities, including amputations [125]. The large number of deaths from snake bites result, in part, from a much larger rate of human–snake encounters. Nevertheless, it is clear that snake envenomations present a significantly larger concern to human health and life, compared to cone snails.

Another large contributor to human envenomations are scorpions, with an estimated 1.2 million global envenomations, and more than 3250 deaths each year [126]. One of the most venomous stings, the eastern red scorpion *Hottentotta tamulus*, has an estimated fatality rate of ≈30% when untreated. Similarly to cone snails, no effective antivenom exists for *H. tamulus* venom, though treatment with the anti-hypertension drug prazosin can lower this fatality rate to 2–4% [127,128].

As with cone snails, other venomous animals have also been an important source of biological, and biomedical research, research tools, as well as drugs and drug leads. Snake venom has provided several clinically important drugs, including blood pressure medication, coagulants, and anticoagulants [129,130,131]. Numerous scorpion venom components are likewise being investigated for biomedical uses, including novel peptide antimicrobial drugs [132].

### 3.2. Fictional Use of Conotoxins as Bioweapons

As envenomation by some species can be deadly, cone snails and their toxins have gained notoriety, both in national biodefense considerations (see Section 3.4), as well as in fiction. Some of these have recently been reviewed elsewhere [121,133].

For instance, in the Michael Crichton novel “The Lost World” (the sequel to “Jurassic Park”), as well as in the movie and video game adaptations of the novel, the “Lindstradt air gun”, a gun shooting a dart containing “enhanced venom” from the cone snail *Conus purpurascens* is used to kill or paralyze dinosaurs. In the movie, *Conus purpurascens* venom is described as the most powerful neurotoxin in the world that acts within 1/2000th of a second, which is stated to be faster than the velocity of nerve conduction.

In a 1972 episode of the television show Hawaii five-0 (season 4, episode 20: “Cloth of Gold”), a *Conus textile*, also called the “cloth of gold”, is intended to be used as a murder weapon. Instead it ends up being used as a tool for suicide by the main antagonist who presses it against his throat and is stung.

The Danish/Swedish television show, “Broen” (“The Bridge”, season 4, episode 5), featured the venom of *Conus geographus* (although an image of *Conus textile* was shown) as a weapon for murder. The toxin used was allegedly manufactured in a conotoxin production facility in Hamburg, Germany.

Another example is an episode of the animated British children’s show “Octonauts” (season 3, episode 3) that featured a cone snail shooting poison-loaded harpoons at crew members after being lost inside an underwater vessel.

Conotoxins have also appeared in several written murder mysteries, such as in James Patterson’s 2018 thriller “Murder in Paradise” or the novel “Murder on the Mataniko Bridge” by Ann Kengalu.

### 3.3. Conotoxin Toxicity

Contrary to their appearance as powerful murder weapons in fiction, no real-life incident for the nefarious use of a cone snail, its venom or toxin components has ever been reported. In this section, we report on the toxicity of some conotoxins in mammals that inspired both their use as weapons in fiction, and the introduction of regulatory measures for scientists working with conotoxins.

Due to the way conotoxins were traditionally identified (i.e., by behavioral bioassays in mice, see Section 1.2), the toxins that are the most potent in mammals were typically among the first to be identified [11,26,27]. As discussed above, these include a toxin from *C. geographus*, α-conotoxin GI, a potent inhibitor of nicotinic acetylcholine receptors of the neuromuscular junction [134,135,136] (Table 4). α-conotoxin GI significantly contributes to the comparably high fatality rate of *C. geographus* envenomations where it is believed to induce muscle paralysis and, ultimately, respiratory arrest due to paralysis of the diaphragm [137]. This toxin was described more than 40 years ago, and yet, to our knowledge, no incidents have ever been reported of its misuse. On the contrary, α-conotoxin GI been a valuable research tool in neurosciences and biochemistry (Table 3). As with all cone snail species, the venom of *C. geographus* contains more than 100 different toxins, the majority of which are not considered harmful to humans. As with α-conotoxin GI, numerous other *C. geographus* toxins have been valuable as drug leads and biomedical tools as well as one diagnostic agent (see Section 2.2, Section 2.3 and Section 2.5).

Since conotoxins comprise a large and diverse class of compounds with many different biomolecular targets in various species, the mammalian toxicity of different conotoxins likewise covers a range of orders of magnitude. The median lethal dose (LD_50_) of α-conotoxin GI is 12 µg/kg when injected intraperitoneally (IP) in mice [11]. Indeed, several conotoxins in the α-conotoxin family that target muscle-type nicotinic acetylcholine receptors of the neuromuscular junction, are quite potent toxins in mammals. However, this group forms a very small subset of α-conotoxins (most target neuronal nAChR subtypes and have very little to no toxicity in mammals) and a minuscule percentage of all conotoxins. For the vast majority of other conotoxins, the toxicity in mammals is so low that no LD_50_ has ever been determined. This not only includes many toxins from worm- or snail hunting species that have little to no effect in vertebrates, but also numerous toxins from fish hunters. For instance, the venom of *C. geographus* contains a vasopressin-like toxin (conopressin-G) that elicits a grooming behavior in mice when injected intracerebrally [24], and insulin-like toxins (coninsulins), that are used by the snail to induce low blood sugar in fish prey but activate the mammalian insulin receptor at much lower potency than human insulin [138]. As stated above, even within the family of α-conotoxins, most toxins have very low to no toxicity in mammals. For instance, α-conotoxin GIC, also from *C. geographus*, targets neuronal nAChRs, does not block human neuromuscular nAChR subunit compositions in electrophysiological assays, nor do mice display any motor deficits or paralysis when injected with up to 5 nmol IP (corresponding to >250 µg/kg) [139].

While certain conotoxins are indeed toxic to humans, these toxins are significantly less potent than certain toxins produced in other animals (see Table 4). For instance, even the most lethal conotoxin is more than one order of magnitude less potent than both textilotoxin, a protein toxin from the eastern brown snake, *Pseudonaja textilis*, as well as ciguatoxins and maitotoxins, which are produced by various dinoflagellate species.

Furthermore, conotoxins appear to only be toxic when injected. While not every route of administration has been described, attempts have been made to improve the oral activity of conotoxin drug leads. An example is the α-conotoxin Vc1.1, where numerous modifications were tested in order to increase its oral bioactivity. The analog obtained with the highest oral bioactivity was still ≈1000 fold less potent when administered orally, than when injected [86,140].

### 3.4. Past and Current Regulations of Research on Conotoxins

Worldwide, various governing bodies are responsible for maintaining lists of regulated substances that are deemed biosecurity concerns. Items on these regulatory lists are subject to certain restrictions in their export and use, including in research. These lists contain various pathogens (e.g., Ebola virus, sheeppox virus) but also include toxins of biological origin. Most of these toxins have a well-defined chemical identity and biological activity, e.g., tetrodotoxin, botulinum toxin, or T2-mycotoxin. However, for conotoxins, this is not the case, and the term “conotoxin” or “conotoxins” is used without additional classification. For example, at the time of writing, the European Union (EU) includes “conotoxin” as a controlled substance [154] and Australian regulations cover “conotoxins” [155], both of which are virtually identical to how the United States regulated conotoxins prior to a 2012 revision. Thus, these two lists not only include conotoxins that have toxicity in vertebrates, but also those that elicit little or no physiological response in vertebrates and those with unknown biological activity. Given the chemical, structural, and biological diversity of conotoxins (see Section 1.1) regulating conotoxins as a single entity is clearly problematic. It is worth noting that to the best of our knowledge, no regulatory agency has ever had “snake toxins” or “scorpion toxins” as a regulated substance in the same manner as “conotoxins”. Neither does any country, to the best of our knowledge, regulate any of the specific components of any animal venoms, even ones that are more potent toxins in mammals than any conotoxin (see Table 4 for examples). Without a clear definition of the term “conotoxin”, as currently the case in many countries, interpretation is often left to the individual evaluating a given case, who is typically not an expert in the field.

To address this, some countries have more narrowly defined their classification of regulated conotoxins. For example, until 2012, the select agent list in the United States included “conotoxins”. This has since been revised to only include the paralytic α-conotoxins containing a very distinct sequence pattern, which corresponds to the sequence motifs found in the conotoxins that block muscle-type nicotinic acetylcholine receptors ([156]; “*Short, paralytic alpha conotoxins containing the following amino acid sequence X_1_CCX_2_PACGX_3_X_4_X_5_X_6_CX_7_ where C = Cysteine residues with the 1st and 3rd Cysteine, and the 2nd and 4th Cysteine forming specific disulfide bridges; X_1_ = any amino acid(s) or Des-X; X_2_ = Asparagine or Histidine; P = Proline; A = Alanine; G = Glycine; X_3_ = Arginine or Lysine; X_4_ = Asparagine, Histidine, Lysine, Arginine, Tyrosine, Phenylalanine or Tryptophan; X_5_ = Tyrosine, Phenylalanine, or Tryptophan; X_6_ = Serine, Threonine, Glutamate, Aspartate, Glutamine, or Asparagine; X_7_ = Any amino acid(s); “Des X” = “an amino acid does not have to be present at this position.”*). This narrower definition was also recently adopted by the Danish Center of Biosecurity and Biopreparedness (CBB) [157].

In 1985, the “Australia Group” was formed as an informal arrangement aimed at allowing members to harmonizing export, while minimizing the risk of this export aiding in chemical and biological weapon proliferation [158]. At the time of writing, the Australia Group has 44 members, including Australia, New Zealand, the United States, Argentina, Mexico, Japan, the Republic of Korea, the United Kingdom, Switzerland, and members of the European Union. Conotoxins are listed as biological agents, thus requiring members to control their international trade with the exception of medical or clinical formulations of conotoxins designated for human use.

The exact implementation of the regulations, in regard to research activities utilizing conotoxins, varies in different countries, but typically researchers are allowed to work with threshold amounts (often 100 mg is used), while being subject to lower regulatory requirements for handling, training, and/or reporting to authorities, whereas higher amounts of conotoxin are typically subject to more stringent restrictions and requirements. Where regulatory agencies have differentiated various conotoxins, this typically applies to a very select group of paralytic and potent toxins in mammals, such as the paralytic α-conotoxins. If differences between conotoxins are not specified, these limitations are typically interpreted to mean that even small amounts of any conotoxin are regulated in this manner, regardless of the toxicity of the specific conotoxin in question.

It is interesting to note that the crude cone snail venom, even from the most venomous species, has never been regulated. Only “conotoxin” components of the venom are regulated, even in cases where the term “conotoxins” is used to encompass every single component of the venom. This is despite the fact that the venom components elicit a synergistic effect, in fact being more potent as crude venom than as the individual components that are regulated [15,159].

### 3.5. Potential Use of Conotoxins as Bioweapons

Although, to our knowledge, there has not been a single incident on the use of cone snail venom or conotoxins outside of legitimate research and drug development programs, the regulatory measures described in the previous Section 3.4 reflect concerns about the potential misuse of conotoxins in bioterrorism.

One such concern is that conotoxins could potentially be aerosolized and thus more easily spread and inhaled by potential victims. The bioavailibilities upon pulmonary inhalation greatly varies between different compounds [160,161,162,163] making it difficult to predict whether any conotoxin would retain toxicity in an aerosolized form. If indeed they did, this would provide an alternate route of administration. However, the toxin would still need to be formulated for aerosolization purposes, and formulating peptides for aerosol delivery is not trivial. Producing the appropriate particle sizes, as well as the being able to retain peptide integrity during the process remains challenging [164].

Another potential concern is that some conotoxins could be injected thereby acting as a murder weapon. However, this also applies to many other biological and non-biological compounds that are lethal when directly injected into the human body, many of which have never been regulated.

The small amount needed for some conotoxins could potentially render them difficult to detect, complicating the determination of the cause of death. The pharmacokinetics of conotoxins in humans are not well described. It has been reported that for α-conotoxin GI, no breakdown was detected after a 3-h incubation in human plasma [165], and for α-conotoxin MII, more than 60% remained after 24 h of incubation in human plasma [166], though the in vivo clearance of these and other conotoxins could be much faster due to metabolism outside of systemic circulation [167]. While modern forensic testing methods are able to detect peptide concentration in plasma of ≈0.1 parts-per-billion [168], it is possible that a conotoxin could metabolize beyond this limit before an autopsy would be performed. A further and likely more pressing concern could be that no antivenom exists. This means that even if a victim could receive care in time, life-saving medical interventions are limited to supportive care (for example, for α-conotoxin GI mouth to mouth or mechanical ventilation can be performed until the paralysis wears off). This, however, is also true for numerous compounds that are not regulated, several of them being more potent than any conotoxin (see Table 4).

Another avenue for potentially using conotoxins as bioweapons could be their incorporation into the genomes of pathogenic viruses and bacteria genomes in order to enhance their deadliness. According to an interview with a former scientist in the United States senate a program of such nature was allegedly carried out in the Soviet Union. This program allegedly led to the generation of a smallpox virus that carried conotoxin sequences before it was ultimately terminated [169]. As stated in the interview these conotoxins contained two specific cystine bridges and, thus, were likely α-conotoxins. While this report could have led to the strict regulations regarding conotoxin research, it should be noted that many other toxins could be used in such a manner and research on dangerous pathogens, including the smallpox virus, is already strictly regulated.

In 2017, El-Aziz and co-workers published a method for in vivo neutralization of toxic peptides using DNA oligonucleotides [170]. As a proof of concept, they used α-conotoxin PrXA from *Conus parius*, a fast acting and potent toxin targeting the nAChRs of the skeletal muscles. They showed that the oligonucleotides (“adaptamers”) could efficiently counteract the binding to receptors, inhibition of diaphragm contraction, and death induced by this conotoxin in mice. While not yet available for clinical use, the World Health Organization has classified envenomations as category A (the highest priority concern available), mostly due to snake bites. Since this approach could also be useful for toxins from other animals, including snakes, these promising efforts could lead to the generation of effective medical treatment options in the future.

## 4. Concluding Remarks

### 4.1. Concluding Remarks on Conotoxin “Cures”

Since the dawn of conotoxin research ≈60 years ago [6,171,172], the number of new conotoxins being identified has exploded. Through the decades, their increasing chemical and pharmacological diversity became apparent and, to date, >5000 research articles have been published in this field of research. Furthermore, conotoxins have been used as tools in thousands of additional research studies, many of which could only be conducted due to the unique properties of certain conotoxins. From a basic understanding of receptor subunit compositions, receptor structures, and peptide folding and expression, to more physiological studies on such diverse topics as epilepsy, inflammation, cancer, pain, cardiology, renal function, and addiction (see Table 3), and even clinical studies and an FDA approved drug (see Table 2), these peptides have already provided immense benefits to basic and applied research and society. With the advances in genomics sequencing, the number of available conotoxin sequences is rapidly increasing. Every new sequence is a new opportunity for furthering research into novel biology, as well as clinical treatments. As long as researchers can use these valuable tools in their research, novel discoveries will continue for many more decades to come.

### 4.2. Concluding Remarks on Conotoxin “Curses”

A few select conotoxins are indeed toxic to humans, but the vast majority are not. It seems self-evident that the harmless conotoxins should not be subject to regulations. However, here we argue that even for the more potent toxins, regulations on researchers are unlikely to prevent their use in bioterrorism, but instead will impede research that, as outlined above, provides many impactful benefits. As we have explained, even the most potent conotoxins appear to be poor candidates for the development of biological weapons (see Section 3.5). Moreover, knowledge of toxin sequences and their synthesis has been publicly available for decades, and the reagents and equipment needed are, to the best of our knowledge, not regulated. In fact, some of these toxins can be readily purchased from commercial providers. However, as discussed, the actual formulation of conotoxins for an aerosol delivery is likely to prove challenging, and it is unclear whether conotoxins would even be bioavailable in such a formulation. With cheap, easy alternatives readily available and proven effective (e.g., phosgene gas), there would seem to be little incentive to pursue this. Consider too, that if successful, recent efforts in developing oligonucleotide-based blockers of peptide toxins may provide broadly applicable treatments. This would further lower the potential of conotoxins as bioweapons. Likewise, concerns about using conotoxins as injectable weapons, while possible, seem largely irrelevant outside of fiction, considering the plethora of other toxins or toxic substances that could easily replace conotoxins in such a scenario.

### 4.3. Suggestions

First, the lack of a clear definition of “conotoxin” or “conotoxins” in legislative work is highly problematic. At the very least, a clear distinction should be made between different conotoxins. If, after a careful consideration of the available literature, any regulatory authority still sees a reason to keep certain conotoxins on the list of potential bioweapon threats, it is essential that these are clearly differentiated from other conotoxins.

Second, it is our opinion that listing even the most potent toxins will have little effect in regard to their potential use in bioterrorism. As discussed, toxin sequences and information on synthesis and recombinant production are publicly available and have been for decades. Limiting the use of these toxins in research is unlikely to reduce a potential bioterror threat. Instead, it is a barrier to research in this important field.

## Figures and Tables

**Table 1:** Pharmacological families of conotoxins (in alphabetical order, modified from [10]).

Pharmacological Family	Molecular Target	Molecular Mechanism	Reference Conotoxin	Reference
α (alpha)	Nicotinic acetylcholine receptors (nAChR)	Receptor antagonists	GI	[11]
γ (gamma)	Neuronal pacemaker cation channels	Channel activator, potentially indirect effect	PnVIIA	[12]
δ (delta)	Voltage-gated Na channel	Delay channel inactivation	PVIA	[13]
ι (iota)	Voltage-gated Na channels	Channel activators	RXIA	[14]
κ (kappa)	Voltage-gated K channels	Channel blockers	PVIIA	[15]
μ (mu)	Voltage-gated Na channels	Channel blockers	GIIIA	[16]
ρ (rho)	α1 adrenoreceptors	Allosteric inhibitor	TIA	[17]
σ (sigma)	5-hydroxytryptamine 3 receptor (HTR3A)	Receptor antagonist	GVIIIA	[18]
τ (tao)	Somatostatin receptor (SSTR)	Receptor antagonist	CnVA	[19]
χ (chi)	Norepinephrine Transporter	Inhibitor	MrIA	[17]
ω (omega)	Voltage-gated Ca channels	Channel blockers	GVIA	[20]
Φ (phi)	Promotes cell proliferation	Not determined	MiXXVIIA	[21]
**Examples of pharmacological families without Greek letter designation**				
Conantokins	N-methyl-D-aspartate receptor (NMDAR)	Receptor antagonists	Conantokin-G	[22]
Coninsulins	Insulin receptor	Receptor agonists	Con-Insulin G1	[23]
Conopressins	Vasopressin receptor	Receptor agonists and antagonists	Lys-Conopressin-G	[24]

**Table 2:** Overview of conotoxin drug leads.

Conotoxin	Molecular Target	Clinical Indication	Stage in Development	Company
MVIIA (ziconotide, Prialt^®^)	Ca_v_2.2 channel	Refractory chronic and cancer pain	Approved	TerSera Therapeutics, Riemser Pharma GmbH, Eisai Co., Ltd.
α-RgIA4 (KCP-400)	nAChR (subtype α9α10)	Neuropathic Pain	Pre-clinical (ongoing)	Kineta, Inc.
Mini-Ins (conotoxin insulin analog)	Insulin receptor	Type 1 diabetes	Pre-clinical (ongoing)	Monolog LLC
Contulakin-G (CGX-1160)	Neurotensin receptor	Neuropathic Pain	Phase I (on hold, demise of company)	Cognetix, Inc.
α-Vc1.1 (ACV1)	nAChR (subtype α9α10)	Neuropathic Pain	Phase I (discontinued, lack of efficacy)	Metabolic Pharmaceuticals
ω-CVID	Ca_v_2.2 channel	Chronic Pain	Phase II (discontinued)	Amrad, Inc.
χ-MrIA (Xen2174)	Norepinephrine transporter	Postoperative pai	Phase II (discontinued)	Xenome, Inc.
Conantokin-G (CGX-1007)	NMDA receptor (subtype NR2B)	Intractable Epilepsy	Pre-clinical (discontinued, demise of company)	Cognetix, Inc.
κ-PVIIA (CGX-1051)	K_v_1 subfamily	Cardioprotection	Pre-clinical (discontinued, demise of company)	Cognetix, Inc.

**Table 3:** Examples of conotoxins used as research tools.

Conotoxin	Target	Feature	Useful in Field(s) of Research
α-GI, μ-SmIIIA, Conantokin-G	Various targets	Substrates for enzymes involved in peptide biosynthesis	Elucidating peptide biosynthesis and folding [68,69,70]
α-ImI	α7 nAChR	Subtype selectivity [56]	Targeted drug delivery in cancer [62], engineering *D. melanogaster* as better human disease model [61], chromaffin cell signaling [57]
α-MII	nAChR	Subtype selective [80]	Inflammation [81], reward and addiction [82,83]
α-Vc1.1 and α-Rg1A	α9α10 nAChR	Subtype selective [84,85]	Neuropathic pain and inflammation [86,87,88], immunology [89,90,91]
Con-ikot-ikot	AMPA receptor	Disrupts desensitization, stabilizes open conformation [63,64]	Receptor crystallization [64]
Con-Insulin G1	Insulin receptor	Minimized binding motif at the insulin receptor [65]	Receptor binding and drug design [92]
κ-PVIIA	Voltage-gated K^+^ channels	Voltage-sensitive binding/blocking of voltage-gated K-channels [15]	Cancer [93], cardioprotection in ischemia [94]
κM-RIIIJ	Voltage-gated K^+^ channels	Subtype selectivity [95,96]	Neuronal profiling [97,98,5,6], channel subtype expression profiling [96,99]
ω-GVIA	Voltage-gated Ca^2+^ channels	Subtype selective [37,99]	Neurotransmission [100,101,102], pain [103], cardiology [55], epilepsy [104], renal function [105], nuclear signaling [106]
ω-MVIIC	Voltage-gated Ca^2+^ channels	Inhibits various subtypes broadly [107,108]	Epilepsy [109], long-term depression [110], pain [111,112]

**Table 4:** Comparison of the median lethal dose (LD_50_) of different toxins and toxic substances.

Toxin	LD50 in Mice (µg/kg)	Route of Administration	Type of Toxin	Source	Known Antivenom/Antidote	Reference
α-conotoxin GI	12	IP	Peptide	*Conus geographus*	No	[11]
ω-conotoxin GVIA	≈60	IP	Peptide	*Conus geographus*	No	[141]
Textilotoxin	1	IP	Protein	*Pseudonaja textilis*	Depends *	[142]
Volkensin	1.38–1.73	IP	Protein	*Adenia volkensii*	No	[143]
Ciguatoxin-1	0.25	IP and oral	Polycyclic poylethers	Various dinoflagellates	No	[144]
Maitotoxin	0.13	IP	Polycyclic poylethers	Various dinoflagellates	No ^†^	[145]
Palytoxin	0.15	IV	Polycyclic poylethers	*Palythoa* corals and dinoflagellates (or bacteria living on these)	No	[146]
Batrachotoxin	2	SC	Alkaloid	Various beetles, birds, and frogs	No	[147]
Saxitoxin	10	IP	Alkaloid	Various marine dinoflagellates	In guinea pigs ^#^	[148]
Tetrodotoxin	8	IV	Alkaloid	Various marine bacteria (e.g., *Pseudoalteromonas tetraodonis*) symbiotically living with numerous marine animals, e.g., *Tetraodontidae* fish, *Hapalochlaena* octopodes, and *Naticidae* snails	No ^†^	[149]

† Supportive treatment provided [150]. * After initial binding phase completed, antivenom seems to have no effect [151,152]. # 4-Aminopyridine (marketed as Ampyra in the US, and used to manage symptoms of multiple sclerosis) has been shown to reverse the effect of saxitoxin poisoning in guinea pigs [153].
